# Tension-induced cytokinetic abscission in human fibroblasts

**DOI:** 10.18632/oncotarget.24016

**Published:** 2018-01-06

**Authors:** Deepesh Kumar Gupta, Jian Du, Siamak A. Kamranvar, Staffan Johansson

**Affiliations:** ^1^ Department of Medical Biochemistry and Microbiology, Biomedical Center, Uppsala University, Uppsala, Sweden; ^2^ First Hospital of Jilin University, Changchun, Jilin, China

**Keywords:** integrin, cytokinesis, abscission, tension, regression

## Abstract

Previous studies have shown that cytokinetic abscission at the end of mitosis is executed by the ESCRT machinery in mammalian cells, and that the process is dependent on adhesion-induced integrin signalling via a FAK-PLK1-CEP55-TSG101/Alix-CHMP4B pathway. The present study identified an alternative abscission mechanism driven by mechanical force. In the absence of integrin signals (non-adherent conditions), cytokinesis in non-transformed human fibroblasts proceeds to CEP55 accumulation at the midbody, but after prolonged time (>3 hours) the major midbody components Aurora B, MKLP1 and CEP55 were no longer detected in the area. Upon adhesion to fibronectin, such cells were able to complete abscission without re-appearance of midbody proteins. Live-cell imaging revealed that re-plating on stiff fibronectin matrix (64 KPa) allowed >95% of the cells to complete abscission within 9 hours while the corresponding number was 40% on soft fibronectin matrix (0.5 KPa). The cells re-plated on poly-L-lysine were not able to generate tension and did not divide. Thus, mechanical tension can cause cytokinetic abscission by stretching of the intercellular bridge between the two daughter cells until it eventually ruptures without the involvement of ESCRT complexes. Importantly, regression of the cleavage furrow and formation of bi-nucleated cells did not occur in most of the suspension-treated mitotic cells after re-plating on fibronectin. Septin, which stabilizes the membrane associated with the midbody, was found to remain along the ingressed membrane, suggesting that this filament system maintains the membrane bridge although the midbody had dissolved, thereby preventing regression and allowing tension to act on the narrow intercellular bridge.

## INTRODUCTION

Proper separation of the cytoplasm between the two emerging daughter cells at the end of mitosis is essential in order to avoid the formation of bi-nucleated cells, since such cells will either die, become tetraploid, or become aneuploid cells in the next mitosis [1]. The highly regulated cytokinesis process starts in early anaphase by formation of the cleavage furrow, and the actomyosin ring-driven ingression of the plasma membrane results in a densely packed bundle of antiparallel microtubules (MT) which allows the assembly of the midbody protein complex [2–5]. Early midbody components, including mitotic kinesin-like protein 1 (MKLP1), Rac GTPase-activating protein 1 (RacGAP), anillin and septin link the structure to the ingressed plasma membrane [6, 7]. After sequential recruitment of several proteins to the midbody, spastin severs the microtubules and the ESCRT III complex eventually drives the process of membrane fusion [8]. Failures at various steps in the cytokinesis process have been reported to cause regression of the cleavage furrow and the generation of bi-nucleated cells [3], a condition known to cause aneuploidy as a hallmark of cancer [9–11].

It is well established that the absence of cell adhesion to extracellular matrix (ECM) causes the cytokinesis process to halt in normal cells [12]. In fibroblasts, integrin-induced signals via FAK and Src is required for the centrosomal protein 55 (CEP55)-mediated binding of endosomal sorting complexes required for transport I (ESCRT I) complex and ALG-2-interacting protein X (ALIX) to the midbody, proteins which in turn recruit the ESCRT III complex [13]. However, mouse fibroblasts cultured in suspension up to 12 hours after karyokinesis were previously found to complete abscission after re-plating on fibronectin, with only a minor fraction instead becoming binucleated [14]. Since the cytokinesis outcome in detached and re-adhering cells may be of relevance for tumourigenesis and tumor progression, we decided to further investigate this issue by closely following the process in the human BJ fibroblasts. The present study shows that the midbody is disassembled within a few hours under non-adherent culture conditions, but in spite of the absence of this structure, regression of the plasma membrane will occur only if the cytokinesis process has not reached to the late stages. Instead cell migration-induced traction force will result in abscission by rupture of the intercellular membrane bridge.

## RESULTS

### The midbody proteins dissociate with time in non-adherent cells

**Figure 1:**
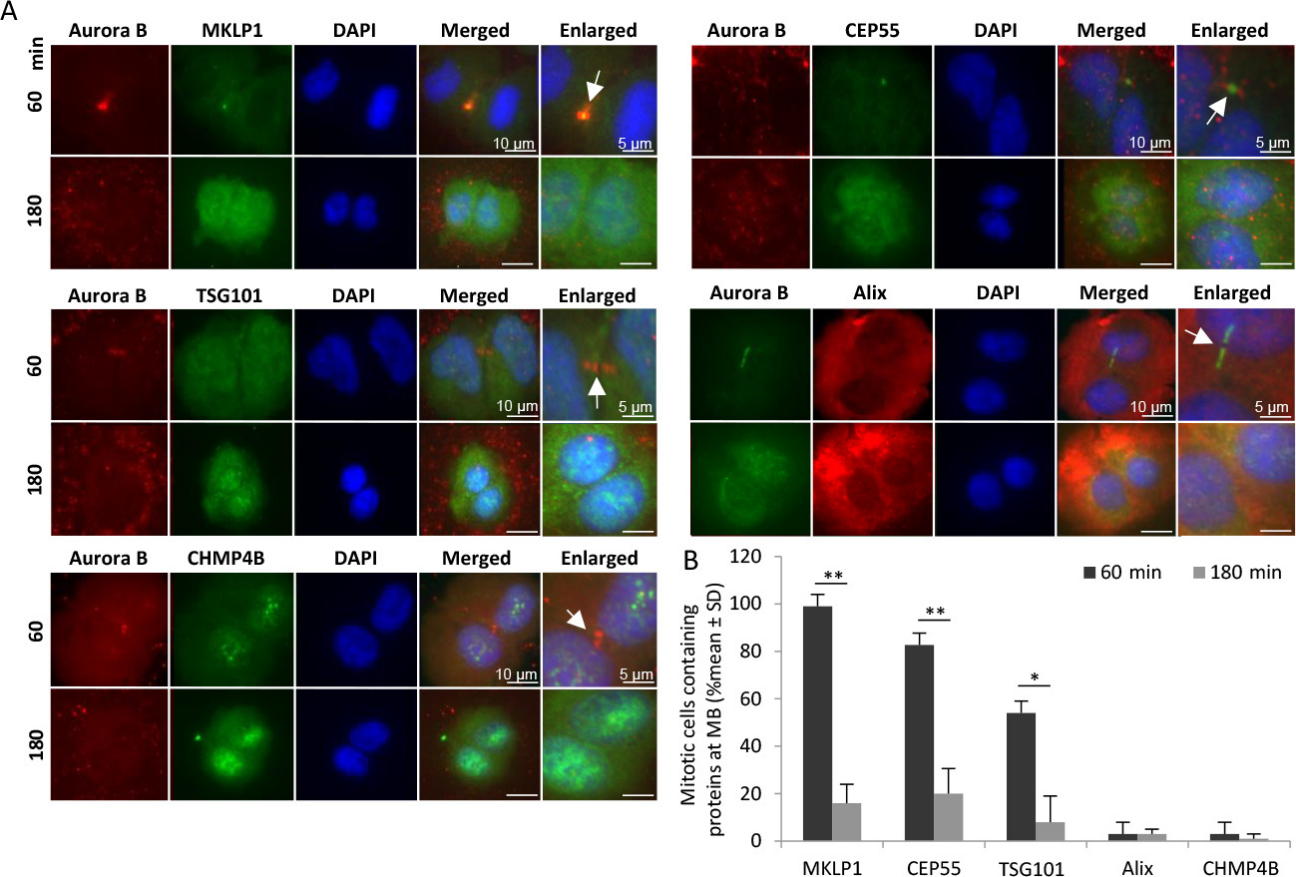
Midbody proteins disappear with time in non-adherent fibroblasts BJ cells in mitotic phase were isolated and incubated in non-adhesive dishes for 60 and 180 minutes. (**A**) Representative immunofluorescence micrographs illustrating the distribution of selected midbody (MB) proteins at the intercellular bridge region (identified by Aurora B labelling; marked by an arrow in the enlarged pictures). Nuclei were stained with DAPI (blue). (**B**) Mean % ± SD of the number of cytokinetic cells containing the analysed proteins in the midbody region.

Our previous studies showed that cytokinesis proceeds in non-adherent human BJ fibroblasts to a stage where CEP55 is present at the midbody and then the process halts [13]. To further investigate the subsequent fate of such cells we closely followed BJ fibroblasts under adherent and non-adherent culture conditions. Immunofluorescent staining of isolated mitotic cells cultured in non-adhesive dishes (Figure 1) showed that the early-phase midbody markers Aurora B and MKLP1 were present at the midbody after 60 minutes in more than 95% of the cells but disappeared after 180 minutes; these proteins are known to have several functions during cytokinesis, including bridging between the midbody and the ingressed membrane by MKLP1. Similarly, CEP55 was localized at the midbody in most of the cells after 60 minutes and disappeared later from this structure. The late-phase midbody proteins ALIX and CHMP4B (an ESCRT III subunit) did not appear at the midbody at any time point in the non-adhesive condition, while ESCRT I subunit TSG101 was present at this site in approximately 40% of the cells after 60 minutes. These data indicate that the midbody that is formed in the absence of adhesion signalling will dissociate over time without completing abscission.

### Detached cells lacking midbody proteins divide after re-adhesion to fibronectin

**Figure 2:**
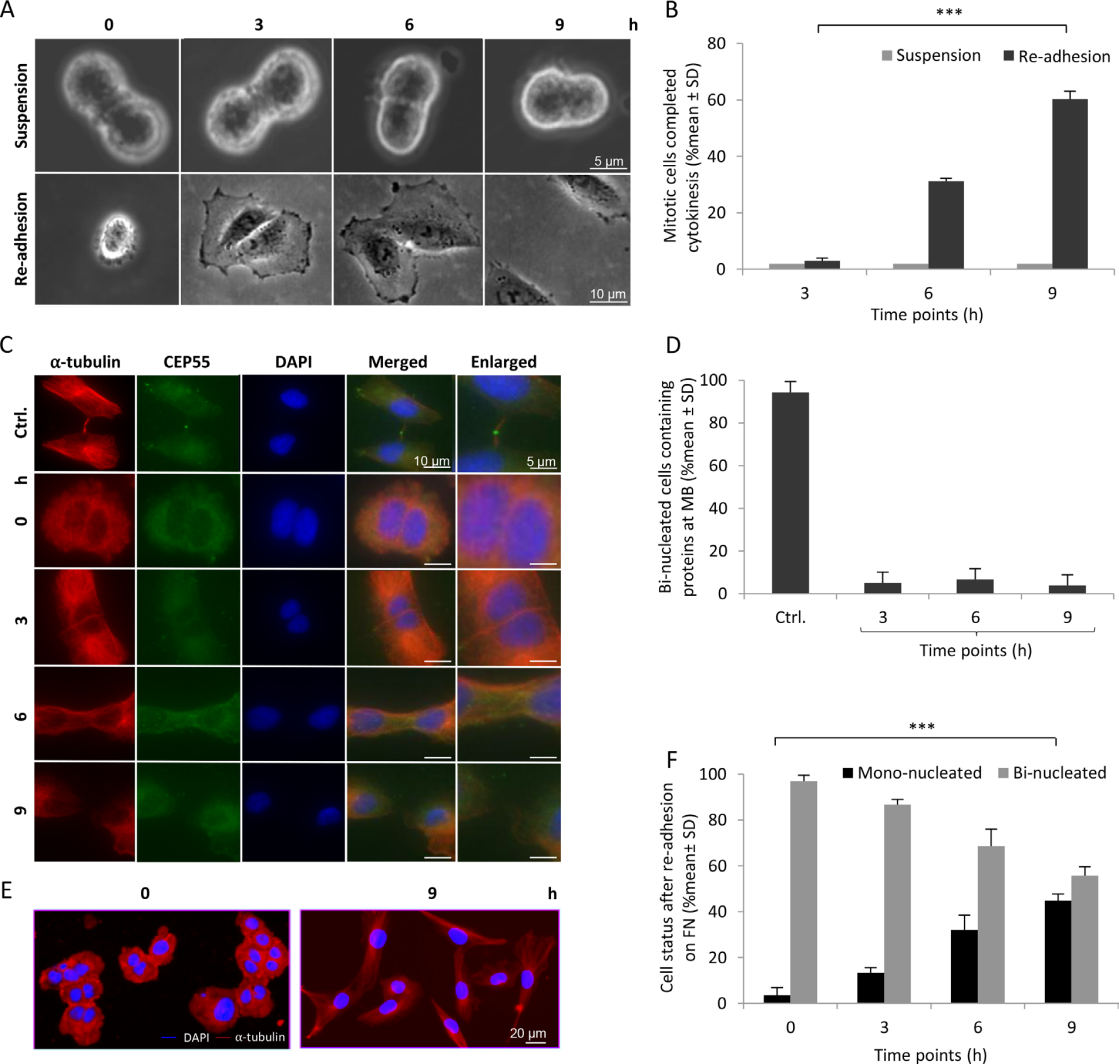
Abscission is completed in the absence of midbody proteins after re-adhesion of 3 hours suspended BJ fibroblasts (**A**) Bright field micrographs from time-lapse movies are illustrating representative single BJ fibroblasts at different time points of cytokinesis; the cells were cultured in non-adhesive dishes (suspension) or in fibronectin-coated dishes (re-adhesion after 3 hours pre-incubation in suspension). (**B**) Mean % ± SD of the mitotic cells completing cytokinetic abscission at the 3, 6, and 9 hour-time points under the conditions described in (A) as analysed by live-imaging. (**C**) Representative immunofluorescence images illustrating the localization of CEP55 (green) in the midbody (MB) regions (labelled with α-tubulin in red) in BJ cells adhering to fibronectin for 0, 3, 6, and 9 hours after a previous incubation for 3 hours in suspension. Nuclei were stained with DAPI (blue). Control cells (Ctrl) were isolated in mitosis phase and directly incubated on fibronectin for 1 hour. (**D**) Mean % ± SD of the number of mitotic cells having CEP55 in midbody at the indicated time points. (**E**) Representative immunostaining images showing mono- and bi-nucleated cells stained for α-tubulin (red) and nucleus (DAPI) at the beginning and 9 h after incubation on FN. (**F**) Mean % ± SD of the mono- and bi-nucleated cells at the 0, 3, 6, and 9 h time points among the cells treated as part (A), analysed by immunofluorescent staining.

The behaviour of the three-hour suspended cells after re-plating on fibronectin-coated dishes was analysed by live cell imaging to allow clear monitoring of any cytokinetic abscission events, the formation of bi-nucleated cells, or cell death. Surprisingly, most of the cells divided, although the completion of the process was delayed (around 60% of the cells divided within 9 hours; Figure 2A, 2B, Supplementary Video 1) compared to normal adherent cells (2–3 hours) [13]. The possibility that the midbody was re-formed in response to adhesion was investigated by staining the cells for key midbody proteins, i.e. CEP55 (Figure 2C, 2D), MKLP1 (Supplementary Figure 1) and ALIX (Supplementary Figure 2), at 3, 6, 9 hours after re-plating on fibronectin; α-tubulin was used as a general midbody marker in these experiments. Notably, CEP55, MKLP1 and ALIX were not detected at any time point in the midbody structure, and α-tubulin was not present as dense MT bundles in the intercellular bridge. The time-lapse data were confirmed by analysing the percentage of mono- and bi-nucleated cells during the same time period after fixation and staining with α-tubulin and DAPI (Figure 2E, 2F). Similar results were obtained by both methods.

### Abscission without midbody depends on the matrix stiffness

**Figure 3:**
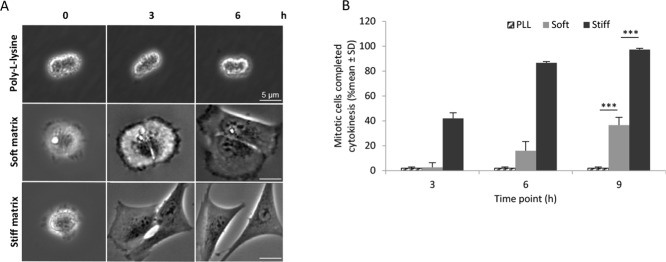
Cytokinetic abscission in the absence of midbody proteins depends on matrix stiffness (**A**) Bright field micrographs from time-lapse movies illustrating representative single BJ fibroblasts at different time points from early-stage cytokinesis; the cells were cultured adhered on poly-L-lysine (PLL) and on soft or stiff matrix conjugated with fibronectin after a previous three-hour incubation in suspension. (**B**) Mean% ± SD of cells with completed cytokinesis under the condition mentioned in (A) as analysed by live-cell imaging.

The live-cell imaging strongly indicated that the abscission-halted daughter cells tried to migrate as individual units and thereby generated traction forces which eventually caused the intercellular bridge to break. To directly test this possible mechanism, suspension-treated mitotic cells were plated on poly-L-lysine (PLL)-coated dishes, as well as on soft (0.5 kPa) and stiff (64 kPa) fibronectin matrices. On PLL, the cells attached but did not spread or migrate and, as expected, abscission did not occur under this condition (Figure 3A, 3B, Supplementary Video 2). The stiff fibronectin matrix efficiently promoted cell migration and abscission in the cells lacking midbody (approximately 85% and 95% divided cells after 6 and 9 hours, respectively) while both processes were markedly slower on the soft fibronectin matrix (approximately 15% and 40% divided cells after 6 and 9 hours, respectively) (Figure 3A, 3B, Supplementary Videos 3 and 4).

### Tension-mediated abscission maintains cell cycle progression

**Figure 4:**
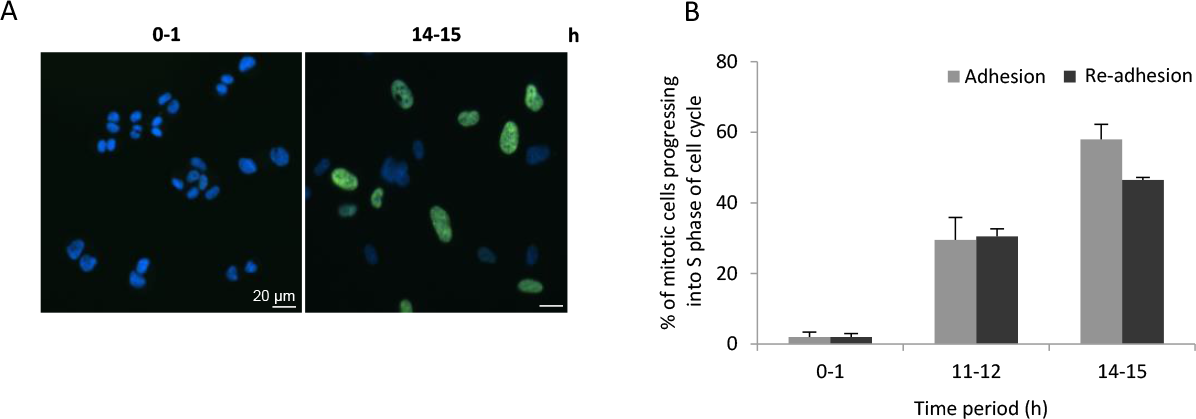
Abscission in the absence of midbody proteins maintains the cell cycle progression (**A**) Representative immunofluorescence images of BJ fibroblasts are illustrating the absence and presence of EdU incorporation in DNA at the indicated time periods of culture on fibronectin after a previous three-hour incubation in suspension. (**B**) Mean% ± SD of the mitotic cells that had progressed into the S phase of the cell cycle at the indicated time points under culture conditions where the cells were continuously adhering to fibronectin or re-plated as described in (A).

To investigate if abscission by mechanical rupture affected the functionality of the cells, the ability to proceed in the following cell cycle into S phase was tested. Mitotic cells were isolated and cultured in suspension for 3 hours as in the previous experiments, plated on fibronectin-coated coverslip for different time periods, and then incubated with the nucleotide analogue EdU during an additional hour on fibronectin. For comparison, mitotic cells continuously cultured on fibronectin was given EdU at the same time periods. In both culture conditions, almost no cells exhibited EdU-labelled nucleus during the first hour after isolation, but after 12 hours (3 hours suspension culture followed by adhesion for 9 hours) the number of EdU-positive cells gradually increased at a similar rate in cells re-plated on fibronectin and continuously adherent control cells (Figure 4A, 4B).

### Regression of cleavage furrow is prevented at late stages of cytokinesis

**Figure 5:**
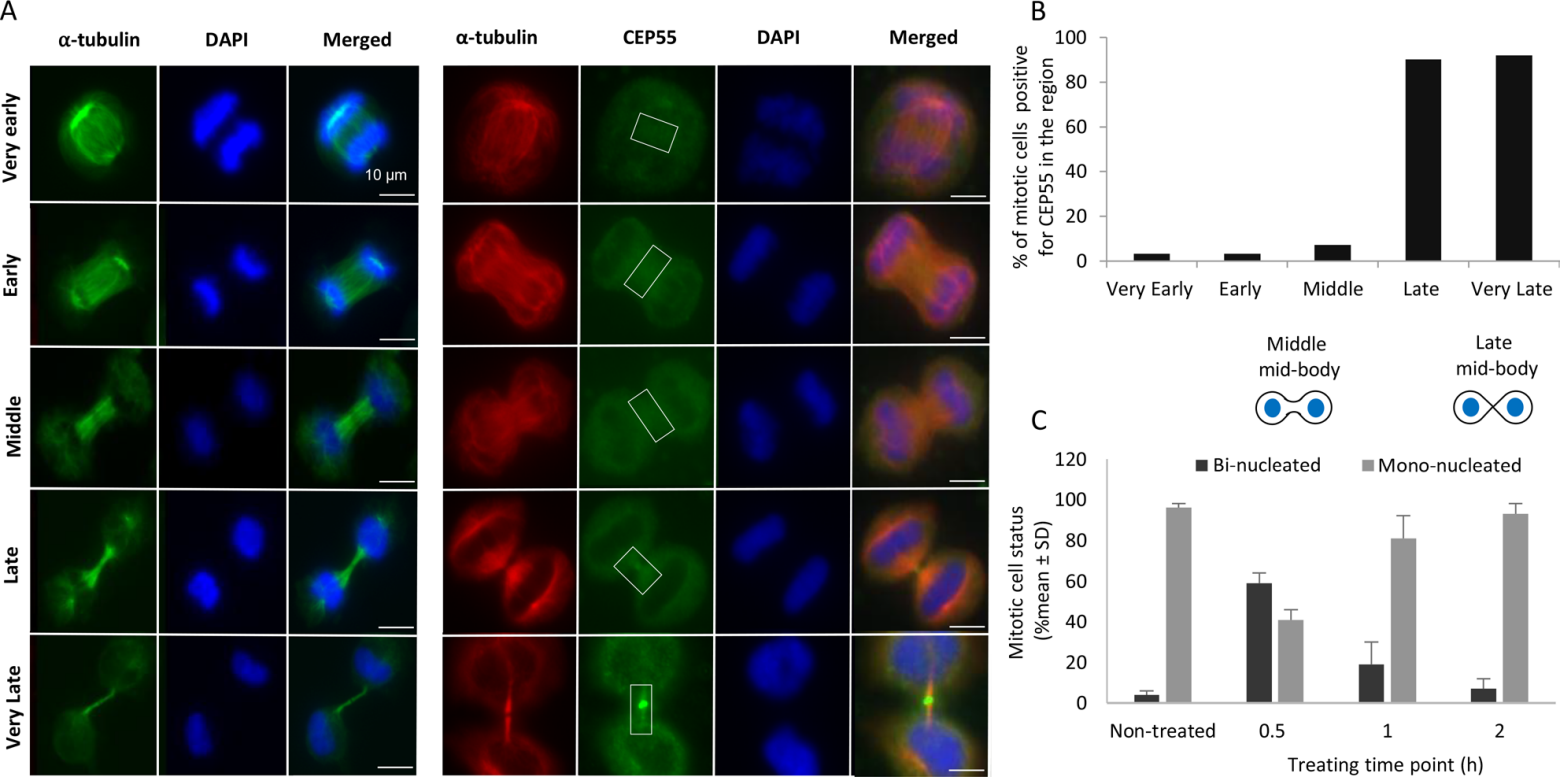
The process of cytokinesis is regressable only before the late furrow ingression phase (**A**) Representative immunofluorescence images indicating the different stages of furrow ingression during cytokinesis. Mitotic BJ cells were immunostained for α-tubulin (green, left panel; red, right panel), CEP55 (green) and nuclei were stained with DAPI (blue). **(B)** % of mitotic cells with CEP55 localisation at the furrow region during different cytokinetic stages as shown in (A). (**C**) Mean % ± SD of mono- and bi-nucleated cells after treatment for 2 hours with cytochalasin D followed by 9 hours without the drug; cytochalasin D was given at 0.5, 1 and 2 hours after isolation of mitotic cells.

After the three-hour suspension period followed by re-plating on fibronectin, the morphology of the cells with two closely located nuclei (Figure 2C, time points 0 h and 3 h) suggested that regression of the cleavage furrow may occur because of the dissolved midbody, and that the traction force later resulting in cells division acted on regressed bi-nucleated cells. However, bi-nucleated cells formed by preventing the initiation of cleavage furrow ingression with cytochalasin D treatment did not undergo abscission by the above described tension mechanism. Initial experiments showed that the adherent cells exposed to 5 μM cytochalasin D for 12 hours followed by 15 hours without the drug became bi-nucleated as expected (Supplementary Figure 3). To avoid a possible effect of long-term cytochalasin D exposure on the traction force from cell migration, a two-hour cytochalsin D treatment to block the furrow ingression followed by incubation without cytochalasin D for 9 hours to allow traction-mediated abscission was tested. A large fraction of the cells (approximately 60%) became bi-nucleated when the cells were exposed to the short cytochalasin D at 30 min after isolation, a time point when the majority of mitotic cells are in early and middle stages of ingression. This number gradually decreased when the drug was given at 1 and 2 hours after isolation, i.e. when the majority of mitotic cells are in late and very late stages of ingression as determined using α-tubulin and CEP55 as markers (Figure 5A–5C). Furthermore, bi-nucleated cells formed by early-stage treatment with cytochalasin D migrated as single units and not as two units trying to migrate independently of each other. These results suggest that regression of the cleavage furrow can occur at initial (very early-middle) stages of cytokinesis, and that regression is prevented when the cytokinesis has progressed to late stages.

### Septin-7 stabilises the ingressed plasma membrane

**Figure 6:**
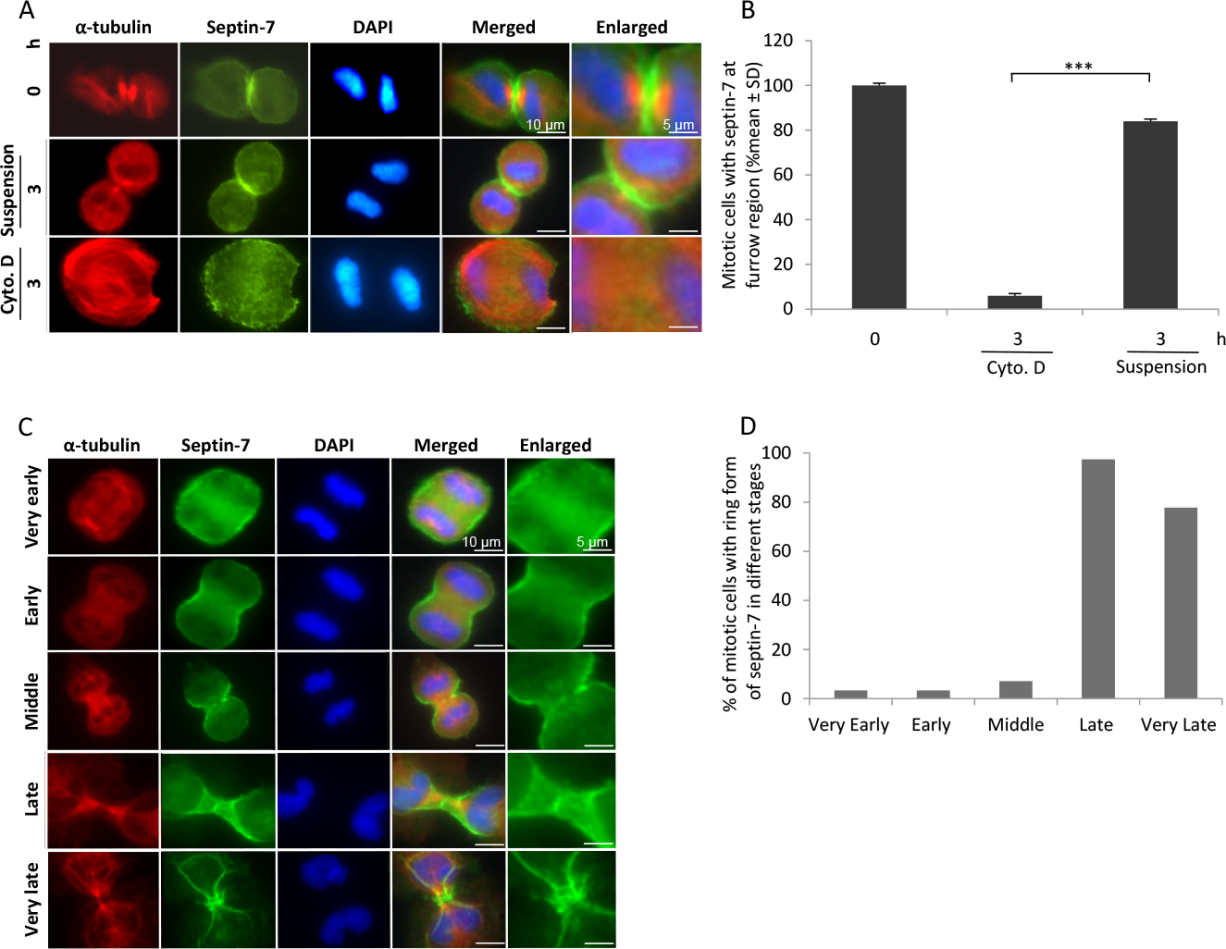
Septin-7 stabilises the ingressed plasma membrane (**A**) BJ cells were synchronized with CDK1 inhibitor and then isolated mitosis cells were incubated on fibronectin for 15 minutes (0 h), in suspension for 3 hours, or on fibronectin for 3 hours in the presence of cytochalasin D. Representative immunofluorescence images showing the distribution of septin-7 (green) and α-tubulin (red). (**B**) Mean% ± SD of the mitotic cells having septin-7 at the cleavage furrow under the different culture conditions described in (A). (**C**) Representative immunofluorescence images illustrating the septin-7 localization during different stages of cytokinesis. Mitotic BJ cells were immunostained for septin-7 (green) and α-tubulin (red), and nuclei were stained with DAPI (blue). (**D**) % of mitotic cells with septin-7 ring structure during different cytokinetic stages as shown in (C).

Since the septin filament system is known to contribute to the stabilisation of the ingressed plasma membrane [15, 16], we analysed if septin may serve the same function also when the midbody had dissolved during suspension culture. Immunofluorescent staining of BJ cells for septin-7 showed that it was enriched at the intercellular bridge and around the midbody in control mitotic cells (Figure 6A, 6B) as previously reported [17]. After suspension culture for 3 hours, septin-7 remained localised along the cleavage furrow, although the midbody proteins had disappeared from this region at the same time. In cells where membrane ingression was inhibited by early cytochalasin D treatment septin-7 was not localised in the mid-zone between the two nuclei (Figure 6A, 6B). The distribution of septin-7 was analysed during different stages of cytokinesis and was found to undergo redistribution from a broad band at the early ingression furrow to concentrated rings around the midbody during late stages (Figure 6C, 6D).

A similar distribution of septin-7 which persisted after dissolution of the midbody structure was observed also in three other analysed cell lines, i.e. 3T6 mouse fibroblasts, mouse embryo fibroblasts (MEFs), and NMuMG mouse epithelial cells. Under non-adherent culture conditions CEP55 disappeared from the midbody region in each one of these cell lines during a three-hour period, and yet, a septin-7 ring was localized at the ingression site (Supplementary Figures 4, 5) and most of the cells divided within 5–9 hours after re-plating on fibronectin in a similar manner as the BJ cells (NMuMG shown in Supplementary Figure 5; 3T6 and MEF not shown). Mitotic cells that were exposed to cytochalasin D for 2 hours directly after isolation and re-plating on fibronectin did not form septin-7 rings in the midzone (analysed after 9 hours as described above for BJ cells).

## DISCUSSION

In contrast to cancer cells, proliferation of normal cells requires the interaction of cells with extracellular matrix. Integrin-mediated adhesion contributes to the cell cycle progression through G1 to S phase and to the completion of the last phase of cytokinesis [18, 19]. A long-standing question has been whether cytokinesis is regulated via specific signals or by mechanical force [2–5, 18, 19]. It is now established that the ESCRT machinery performs cytokinetic abscission after its recruitment to the midbody by a highly regulated process. We recently reported that an integrin-induced signalling pathway involving FAK, PLK1 and CEP55 is required for the binding of the ESCRT components TSG101 and ALIX to the midbody [13]. In addition, mechanical tension has been implicated to regulate cytokinesis in different ways. Traction force acting on the intercellular bridge has been reported both to promote [20] and to delay abscission [21]. Moreover, dermal fibroblast, but not mesenchymal stem cells, were found to require a stiff ECM to divide [22]. Since integrins can generate different signals by ligand-induced clustering and by mechanical tension [23] they may contribute to cytokinesis by several mechanisms, which may be a source for the diverging observations.

**Figure 7:**
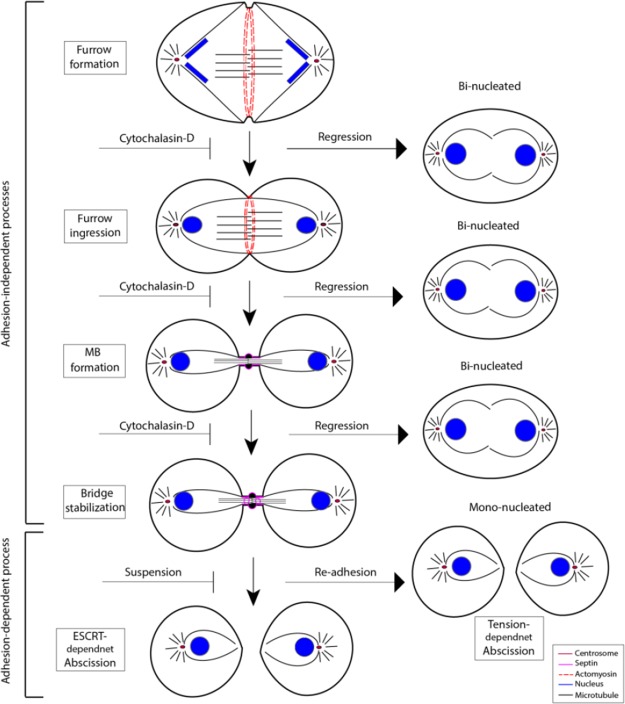
A model describing the effects of cytochalasin D and adhesion on cytokinesis and furrow regression The cytokinesis process is regressable after cytochalasin D treatment until the late stage in which the intercellular bridge is stabilized, possibly by septin filaments; the regression results in the generation of bi-nucleated cells. In cells where the normal ESCRT-mediated abscission fails, abscission can still be completed by migration-mediated traction on the intercellular bridge.

The results of our present study clarifies some of the contradicting observations in previous reports. Separation of mammalian cells by traction force exerted on the intercellular bridge by the migration of the two daughter cells has previously been observed [20, 24], but here we demonstrate that such unconventional cell division occurs at high frequency under certain condition, i.e. in re-adhering cells after previous detachment. Since traction force is dependent on the stiffness of the adhesive surface, the efficiency of this abscission mechanism is promoted by stiff matrices. The mechanism does not require the presence of a midbody and occured only if cytokinesis had developed to a late stage. Normally, the late phase of cytokinesis is rapidly completed via the ESCRT III-dependent abscission mechanism, but in case it fails, a maintained intercellular bridge between the daughter cells will prevent membrane regression and allow efficient tension-induced abscission (Figure 7). More than 80% of such cells divided within 6 hours when cultured on stiff fibronectin surfaces, and >90% had divided after 9 hours. In the absence of cleavage furrow ingression, or when furrow regression occurred, the bi-nucleated cells migrated as single units instead of two units migrating independently of each other. Midbody-independent fission of binucleated cells after blocking the initiation of furrow ingression by blebbistatin or cytochalasin D in human epithelial cells was recently reported. This phenomenon was found to occur in 2% of the cells in each cell cycle [24], and thus, it is a rare event in agreement with our results. Why and how some cells undergo such “cytofission” in the apparent absence of membrane ingression remains unknown.

The critical step in midbody maturation that has to be reached in order to prevent regression is presently not known, but our data indicates that the organisation of septin filaments may be a key factor to keep the plasma membrane ingressed without midbody proteins. During the late stages of cytokinesis, the septin distribution was altered from a broad and longitudinal to narrow and perpendicular form relative to the intercellular bridge (Figure 6). This is reminiscent of the reorganisation of septin filaments during cytokinesis in budding yeast [25]. Formation of perpendicular rings of anillin-septin complexes has also been described in HeLa cells [26]. In our study, the perpendicular septin distribution remained even when midbody proteins were no longer detected (Figure 6A, Supplementary Figures 4, 5) and may thus have prevented furrow regression. In support of this conclusion, key roles of septins in the cytokinesis process and for the stability of the intercellular bridge have been demonstrated in several excellent studies [15, 25–27]. However, the understanding of the septin filament system is still limited and two important questions for understanding when furrow regression and bi-nucleation will occur are: how is the formation of different types of high-order septin structures (linear filaments, rings, gauzes) regulated and how are the structures depolymerized? Recent reports indicate that these events are regulated by complex modifications of septins including phosphorylations and sumoylations [28–30], but further studies are clearly warranted on the regulation of septins and their role in cleavage furrow stabilisation.

Since cleavage furrow regression after failed cytokinesis will result in cells with more than two centrosomes in the following mitosis, it is a potential cause for the formation of aneuploid tumorigenic cells. Cleavage furrow regression has therefore been a topic for studies in numerous reports. However, since the intracellular cytokinetic bridge is narrow, it is often not obvious if two closely located nuclei represent two divided but interacting cells, one bi-nucleated cell with ingressed plasma membrane, or one bi-nucleated cell with regressed plasma membrane. When adherent non-confluent cells are studied *in vitro*, time-lapse recording of live cells is one method which allows clear determination of this issue, but other methods are needed for studies of non-adherent cells or closely associated cells *in vitro* and *in vivo*. Our results suggest that the localisation of septin may be a useful marker for the analysis of abscission and regression.

## MATERIALS AND METHODS

### Cell lines and culturing of mitotic cells

Human non-transformed fibroblasts (hTERT-immortalized BJ cells), mouse fibroblasts (3T6 and SV40LT-immortalized wt MEFs [31], and mouse epithelial cells (NMuMG) were cultured in Dulbecco’s modified Eagle medium (DMEM, Gibco, Life technologies, UK) supplemented with 10% fetal bovine serum (FBS, FB-1090–500, Werner Saveen, Biological Industries, Beit-Haemek Ltd, Israel), 100 U/ml penicillin and 0.1 mg/ml streptomycin (complete medium). The cells were kept at 37° C in a humid atmosphere containing 5% CO_2_. In some experiments CDK1 inhibitor RO-3306 (Sigma-Aldrich, Saint Louis, USA) and cytochalasin D (Sigma-Aldrich) were used to synchronize the cells at the G2-M phase transition.

Mitotic cells were collected by the shake-off method [32] in which exponentially growing cells were washed once with pre-warmed PBS followed by incubation in the complete medium for approximately 3 hours after which the loosely attached mitotic cells were detached by tapping the culture flasks. The suspended mitotic cells were then collected by centrifugation and re-suspended in fresh complete medium for the culturing in plates coated with either the non-adhesive polymer Pluronic (10 mg/ml, F108, D-BASF, Germany), the integrin ligand fibronectin (40 μg/ml), or the non-specific adhesive ligand poly-L-lysine (PLL, 100 μg/ml). In some experiments, surfaces with different stiffness (SoftSubstrates™, MuWells, San Diego, USA) were used after covalent coupling of fibronectin to the surface as described by the manufacturer.

### Live-cell imaging

Live-cell imaging was performed using an inverted microscope (Nikon-Eclipse Ti-U, Japan) equipped with a CCD camera (Andor’s multi pixel sCMOS camera, Oxford Instruments) and a cell culture chamber having constant supply of humidified 5% CO_2_ and temperature control. The images were acquired using an automated motorized multi-position stage with 20× and 40× magnification objectives and phase contrast filter of the time-lapse microscope in 1 or 5-minutes time intervals for the desired time periods.

### Immunofluorescence staining

For the adhesive condition, the mitotic cells were cultured on fibronectin-coated glass coverslips, whereas for the non-adhesive condition they were cultured in Pluronic-coated 10 cm bacterial plates (Sarstedt, Sweden) and thereafter the cells were deposited on glass slides by cytospin centrifugation. Subsequently, the cells were fixed by cold methanol at −20° C for 20 minutes and then washed twice in PBS for 5 minutes. After incubation in blocking buffer containing 1% BSA (Fraction V Roche Diagnostic, Germany) and 0.1% Tween20 (Merck, Germany) in PBS, the slides were incubated overnight at 4° C with primary antibodies at a 1:50 dilution in blocking buffer. Antibodies directed against the following proteins were used: Aurora B (ab-2254 and ab-3609, Abcam, Cambridge, UK), CEP55 (sc-134622 and sc-37405, Santa Cruz, California, USA), α-tubulin (T6199, Sigma, Saint Louis, USA), MKLP1 (sc-22793, Santa Cruz), Septin-7 (JP18991, IBL International, Hamburg, Germany). The slides were then washed with PBS and incubated for 1 hour with a 1:500 dilution in blocking buffer of secondary antibodies, Alexa Fluor 488-conjugated goat anti-rabbit and Alexa Fluor 594-conjugated goat anti-mouse (Invitrogen, Carlsbad, USA), followed by washing in PBS and mounting with medium containing DAPI (4,6-diamidino-2-phenylindole, Invitrogen). Digital images of the cells were captured using a Nikon fluorescence microscope (Nikon Eclipse 90i, Japan) equipped with a CCD camera (DS-Qi1 Monochromatic Digital Camera). The digital images were analysed for the presence or absence of immunostained proteins at the midbody and scored using Adobe Photoshop^©^ (Adobe Photoshop CS6, Adobe system Inc. San Jose, CA, USA) and ImageJ (http://rsb.info.nih.gov) software.

### Quantification of cytokinesis failure

Upon plating on fibronectin-coated substrate, the isolated round mitotic cells flatten, divide, and migrate away from each other. Successful cell division could be clearly identified when they had moved apart. In the fixed samples, the cells were scored as bi-nucleated when two cell bodies were in contact. Since the migrating cells frequently made transient contacts with each other, live-cell imaging allowed a more accurate analysis.

### EdU incorporation analysis

EdU detection was performed according to the protocol (Click-iT™ EdU Imaging Kit, C10084, Invitrogen Molecular Probes, Eugene, Oregon, USA). The cells were incubated with 10 μM EdU for 1 hour prior to fixation with 4% formaldehyde and fluorescence microscope imaging to analyse EdU incorporation.

### Statistical analysis

The statistical analyses were performed using student’s *t*-test. *P*-values < 0.05 were considered as significant. For all the experiments, 50 randomly selected cells per condition and for each time point were analysed from each of three independent experiments. ***, **, and * represent *P*-value less than 0.001, 0.01 and 0.05, respectively.

## SUPPLEMENTARY MATERIALS FIGURES AND TABLES










