# Supplementary material for: Tension-induced cytokinetic abscission in human fibroblasts
Source: Oncotarget. 2018 Jan 6;9(10):8999–9009. doi: 10.18632/oncotarget.24016 (PMC5823655; doi:10.18632/oncotarget.24016)
Supplement: Supplementary file 1 [file oncotarget-09-8999-s001.pdf]

Tension-induced cytokinetic abscission in human fibroblasts

SUPPLEMENTARY MATERIALS

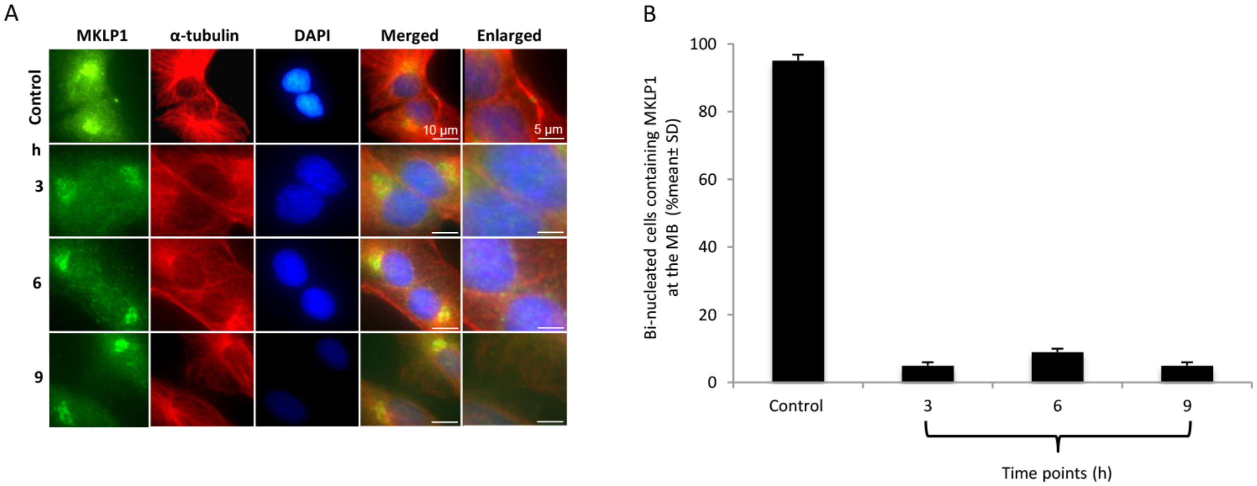

Supplementary Figure 1: MKLP1 disappears with time from the midbody in non-adherent fibroblasts. (**A**) Representative immunofluorescence images illustrating the localization of MKLP1 (green) and α-tubulin (red) in BJ cells adhering to fibronectin for 3, 6, and 9 hours after a previous incubation for 3 hours in suspension upon isolation at the mitosis phase. Nuclei were stained with DAPI (blue). (**B**) Mean % ± SD of the number of cytokinetic cells containing MKLP1 at the midbody (MB).

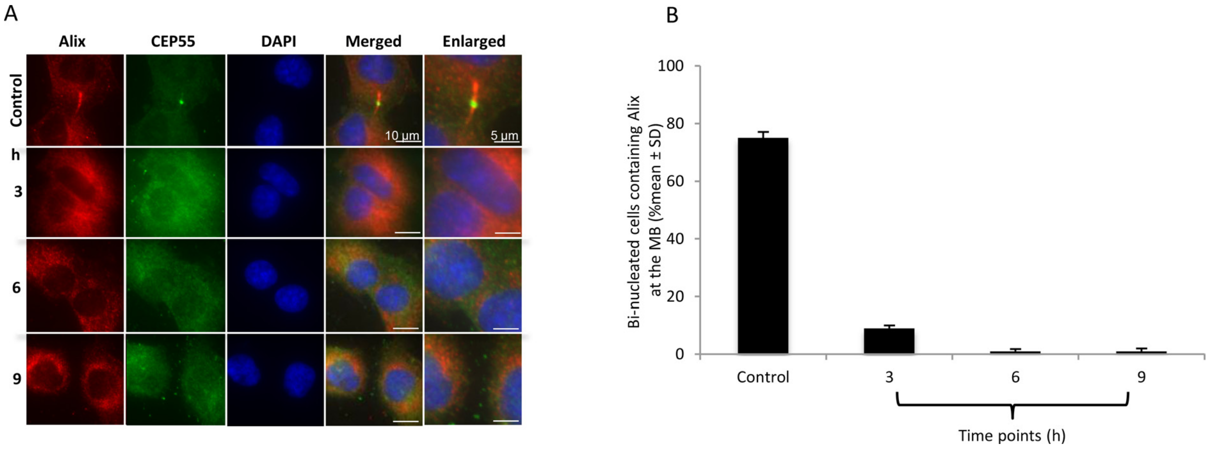

Supplementary Figure 2: ALIX disappears with time from the midbody in non-adherent fibroblasts. (**A**) Representative immunofluorescence images illustrating the localization of ALIX (red) and CEP55 (green) in BJ cells adhering to fibronectin for 3, 6, and 9 hours after a previous incubation for 3 hours in suspension upon isolation at the mitosis phase. Nuclei were stained with DAPI (blue). (**B**) Mean % ± SD of the number of cytokinetic cells containing ALIX at the midbody (MB).

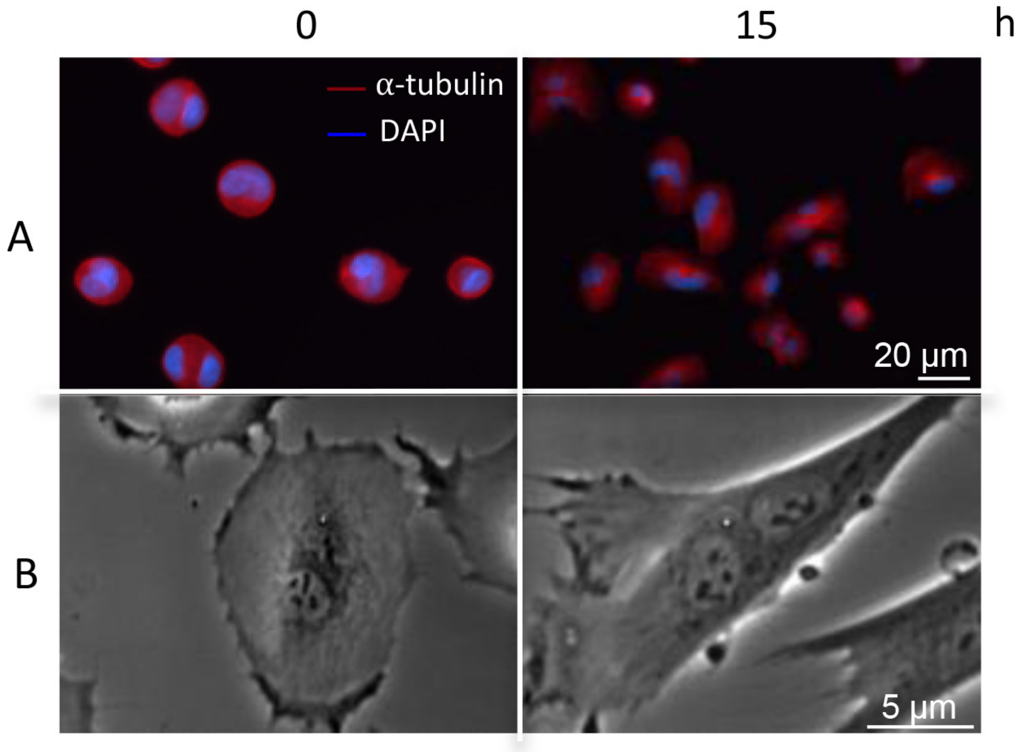

Supplementary Figure 3: Cytochalasin D prevents the ingression of cleavage furrow. Immunofluorescence (**A**) and bright field micrographs (**B**) illustrating the fate of a mitotic cell after treatment with cytochalasin D for 12 hours followed by 0 and 15 hours without the drug.

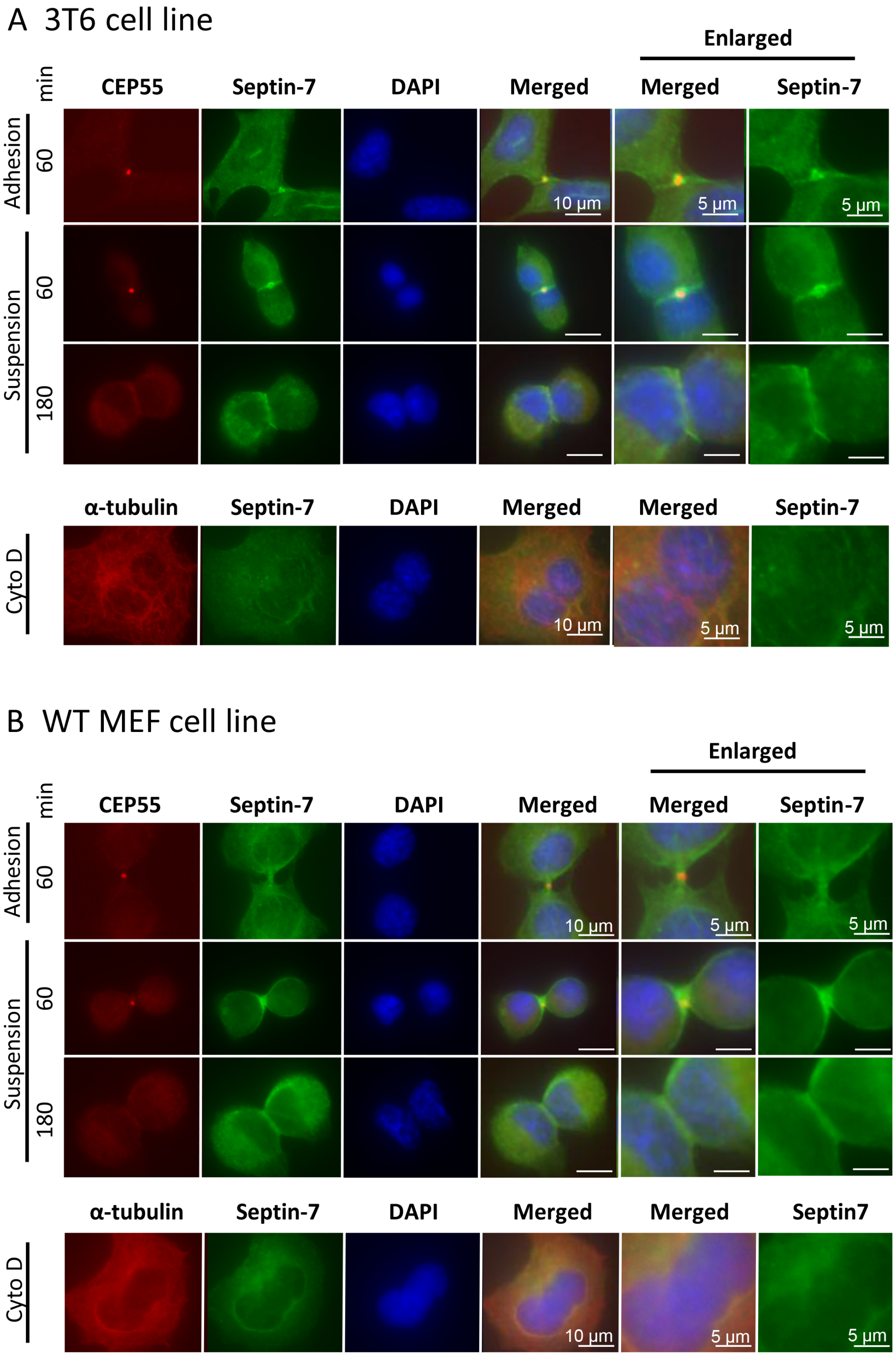

Supplementary Figure 4: Septin-7 stabilizes the cleavage furrow in the abscence of midbody proteins in mouse fibroblast cell lines: (**A**) 3T6, (**B**) MEF. Representative immunofluorescence micrographs illustrating the localization of septin-7 (green) in the midbody region (also stained for CEP55 or α-tubulin, red) after incubation of mitotic cells on fibronectin for 60 min, in suspension for 60 min or 180 min, and on fibronectin in the presence of cytochalasin D (5 µM) for 120 min, respectively. Nuclei were stained with DAPI (blue).

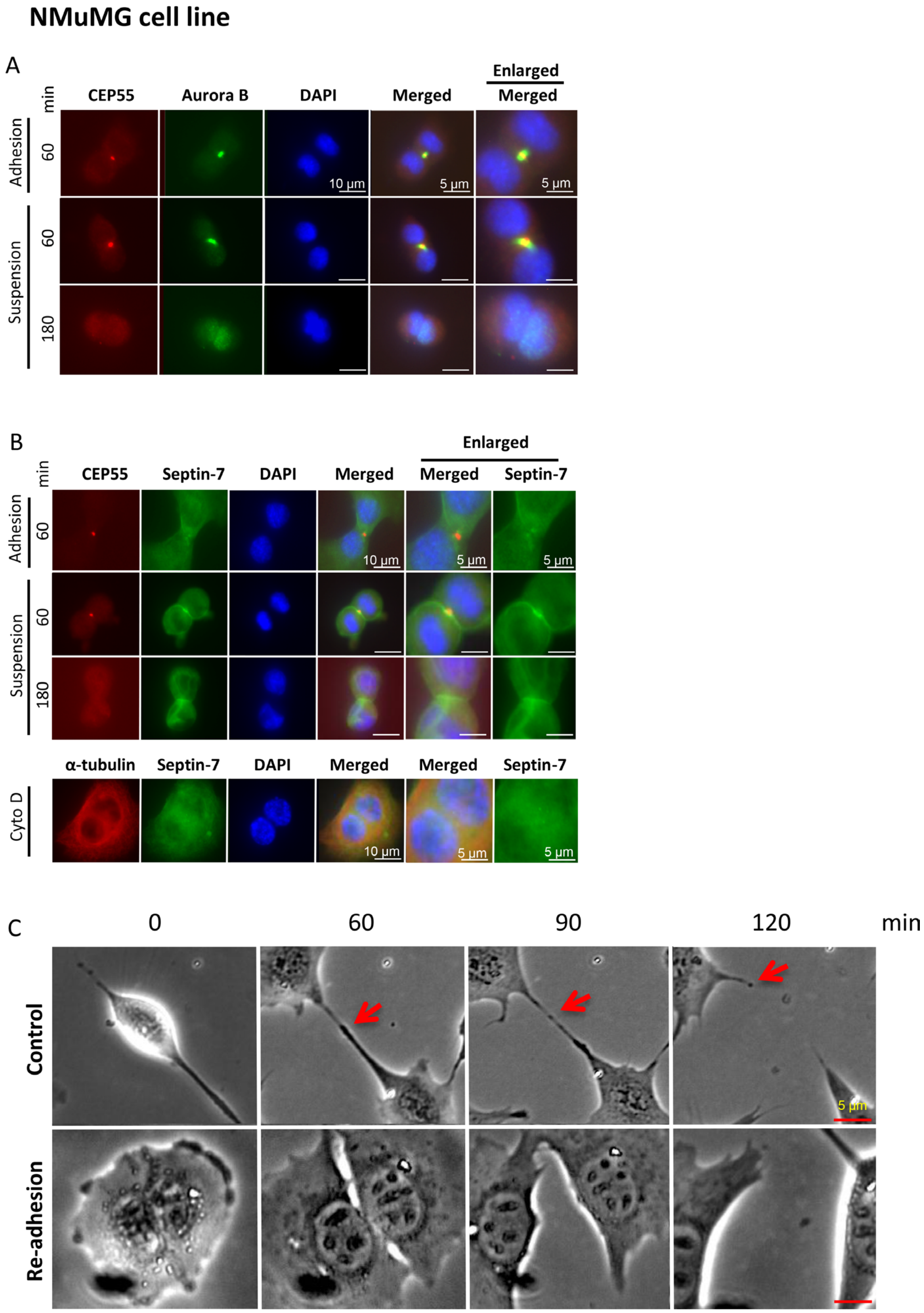

Supplementary Figure 5: Abscission is completed in the absence of midbody proteins after re-adhesion of three-hour suspended NMuMG epithelial cells. NMuMG cells in mitotic phase were isolated and incubated on fibronectin for 60 min or in suspension for 60 min and 180 min. (**A**) Representative immunofluorescence micrographs illustrating the distribution of CEP55 (red) and Aurora B (green). Nuclei were stained with DAPI (blue). (**B**) Representative immunofluorescence micrographs illustrating the localization of septin-7 (green) in the midbody region (also stained for CEP55 or α-tubulin, red) after incubation of mitotic cells on fibronectin for 60 min, in suspension for 60 min or 180 min, and on fibronectin in the presence of cytochalasin D (5 µM) for 120 min, respectively. Nuclei were stained with DAPI (blue). (**C**) Representative bright field micrographs from time-lapse movies of single NMuMG cells progressing through cytokinesis to abscission. Mitotic cells were re-plated in fibronectin-coated dishes either directly after isolation (control) or after a three-hour period in suspension. Arrows mark the midbody in the control cells.

Supplementary Video 1: Cytokinetic abscission in the absence of midbody: BJ fibroblasts showing the progression of cytokinesis during 9 hours on fibronectin-coated tissue culture plate after a previous three-hour suspension incubation in non-adhesive Pluronic-coated dish. See Supplementary_Video_1

Supplementary Video 2: Cytokinesis process on low adhesion surface: BJ fibroblasts isolated at the mitosis phase were monitored during 9 hours on poly-L-lysine-coated ultra-low attachment plate after a previous three-hour incubation in suspension. See Supplementary_Video_2

Supplementary Video 3: Cytokinetic abscission on soft fibronectin matrix: BJ fibroblast isolated at the mitosis phase were monitored during 9 hours on fibronectin-conjugated soft matrix (0.5 kPa) after a previous three-hour incubation in suspension. See Supplementary_Video_3

Supplementary Video 4: Cytokinetic abscission on stiff fibronectin matrix: BJ fibroblast isolated at the mitosis phase were monitored during 9 hours on fibronectin-conjugated stiff matrix (64 kPa) after a previous three-hour incubation in suspension. See Supplementary_Video_4
